# Balanced polymorphisms and their divergence in a *Heliconius* butterfly

**DOI:** 10.1002/ece3.8423

**Published:** 2021-12-08

**Authors:** James G. Ogilvie, Steven Van Belleghem, Ryan Range, Riccardo Papa, Owen W. McMillan, Mathieu Chouteau, Brian A. Counterman

**Affiliations:** ^1^ Department of Biological Sciences Auburn University Auburn Alabama USA; ^2^ Department of Biology University of Puerto Rico Rio Piedras Puerto Rico; ^3^ Smithsonian Tropical Research Institute Panama City Panama; ^4^ Laboratoire Écologie, Évolution, Interactions des Systèmes Amazoniens (LEEISA) Université de Guyane CNRS IFREMER Cayenne French Guiana

**Keywords:** diversification, *Heliconius*, Müllerian mimicry, polymorphic mimicry, polymorphism

## Abstract

The evolution of mimicry in similarly defended prey is well described by the Müllerian mimicry theory, which predicts the convergence of warning patterns in order to gain the most protection from predators. However, despite this prediction, we can find great diversity of color patterns among Müllerian mimics such as *Heliconius* butterflies in the neotropics. Furthermore, some species have evolved the ability to maintain multiple distinct warning patterns in single populations, a phenomenon known as polymorphic mimicry. The adaptive benefit of these polymorphisms is questionable since variation from the most common warning patterns is expected to be disadvantageous as novel signals are punished by predators naive to them. In this study, we use artificial butterfly models throughout Central and South America to characterize the selective pressures maintaining polymorphic mimicry in *Heliconius doris*. Our results highlight the complexity of positive frequency‐dependent selection, the principal selective pressure driving convergence among Müllerian mimics, and its impacts on interspecific variation of mimetic warning coloration. We further show how this selection regime can both limit and facilitate the diversification of mimetic traits.

## Graphical abstract

The selective pressures maintaining polymorphism among Müllerian mimics are relatively unknown. Here, we use artificial butterfly models to characterize how regional differences in predation pressures can both drive and dissuade the evolution of warning color patterns in *Heliconius* butterflies.

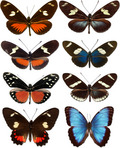

## 1 INTRODUCTION

The diversity of color patterns found in the *Heliconius* butterfly radiation is a striking example of the power of natural selection to generate biodiversity. However, while the most popular theory describing the evolution of these vivid color patterns proposes a framework dissuading from wing pattern diversity, we in fact find dozens of established color patterns throughout the neotropics (Joron & Mallet, 1998; Mallet & Joron, 1999; Moest et al., 2020; Müller, 1879).

Franz Müller (1879) suggested in his theory that mimicking organisms which are unpalatable, venomous or toxic to predators, benefit from reduced predation by converging on common warning patterns. As these organisms become all the more similar over time, Müllerian mimicry theory predicts that the weight of predation will be optimally shared among the mimicking populations. Furthermore, the evolution of stark warning colorations (aposematism) increases the effectiveness of this evolutionary strategy by providing memorable patterns and colors to predators (Su et al., 2015). Examples of animals that through natural selection have trodden this evolutionary journey are familiar to many of us for their striking aspects (e.g., pit vipers, poison dart frogs, bumblebees, and wasps (Sanders et al., 2006; Symula et al., 2001; Williams, 2007; Boppré et al., 2016)). The main mechanism driving this mimicry is known as positive frequency‐dependent selection (pFDS), where the most common warning signal is more likely to spread through a population as it will be most avoided by predators (Müller, 1879). In the past decades, empirical evidence has largely validated pFDS to be a principal selective force maintaining such phenotypic convergence throughout the animal kingdom (Borer et al., 2010; Chouteau et al., 2016; Dumbacher & Fleischer, 2001; Mallet & Barton, 1989; Miller & Pawlik, 2013; Noonan & Comeault, 2008; Sanders et al., 2006; Symula et al., 2001).

*Heliconius* butterflies are a renowned example of Müllerian mimicry. However, as first described by Henry Walter Bates (1862), the genus clearly demonstrates a diverse array of warning color patterns established throughout several mimicry rings. This presents a challenge to Müller's theory which predicts that the selective pressures enacted by predators attacking novel color patterns should force the convergence of many warning signals into few easily recognizable color patterns. In contrast to this expectation, the co‐mimics *Heliconius erato* and *Heliconius melpomene* diverged into over 25 geographic color pattern morphs (Bates, 1862; Mallet and Gilbert, 1995; Turner, 1975; Van Belleghem et al., 2020). These mimicry rings maintain homogenous local warning color patterns within their borders through localized pFDS mostly driven by a few insectivorous birds such as rufous‐tailed jacamars and tyrant flycatchers (Benson, 1972; Chai, 1986; Langham, 2004; Mallet & Barton, 1989; Pinheiro, 2011). However, at the boundaries of these mimicry rings hybridization frequently occurs and results in narrow regions of intermediate color patterns (Edelman et al., 2019; Mallet, 1986a; Thurman et al., 2019). Such phenomena can also be observed in vertebrate Müllerian mimics such as the dendrobatid poison dart frog radiation (Roland et al., 2017).

In contrast to the homogenous local warning color patterns, some species have evolved the ability to maintain multiple mimetic warning phenotypes in a single population, a phenomenon known as “polymorphic mimicry” (O'Donald & Pilecki, 1970). In these populations, distinct morphs are locally adapted to their environment by sharing distribution with other Müllerian co‐mimics (Arias et al., 2016). The selective pressures that allow polymorphic mimicry to evolve and be maintained remains a largely unresolved question. Historically, polymorphy was considered to be a random occurrence with no obvious advantages to the organism bearing it. However, initial evidence in banded land snails (Cain & Sheppard, 1954) and later in a variety of other organisms such as spiders, guppies, and wolves (Hedrick et al., 2016; Hendrickx et al., 2015; Hughes et al., 2013), has indicated that polymorphism may serve an adaptive role that can be maintained through sexual selection and possibly promote speciation (Jamie & Meier, 2020). Such a system has been described in *Heliconius numata*, where polymorphism is considered as the result of competing selective pressures on the genomic architecture underlying the trait (Jay et al., 2021).

**FIGURE 1:**
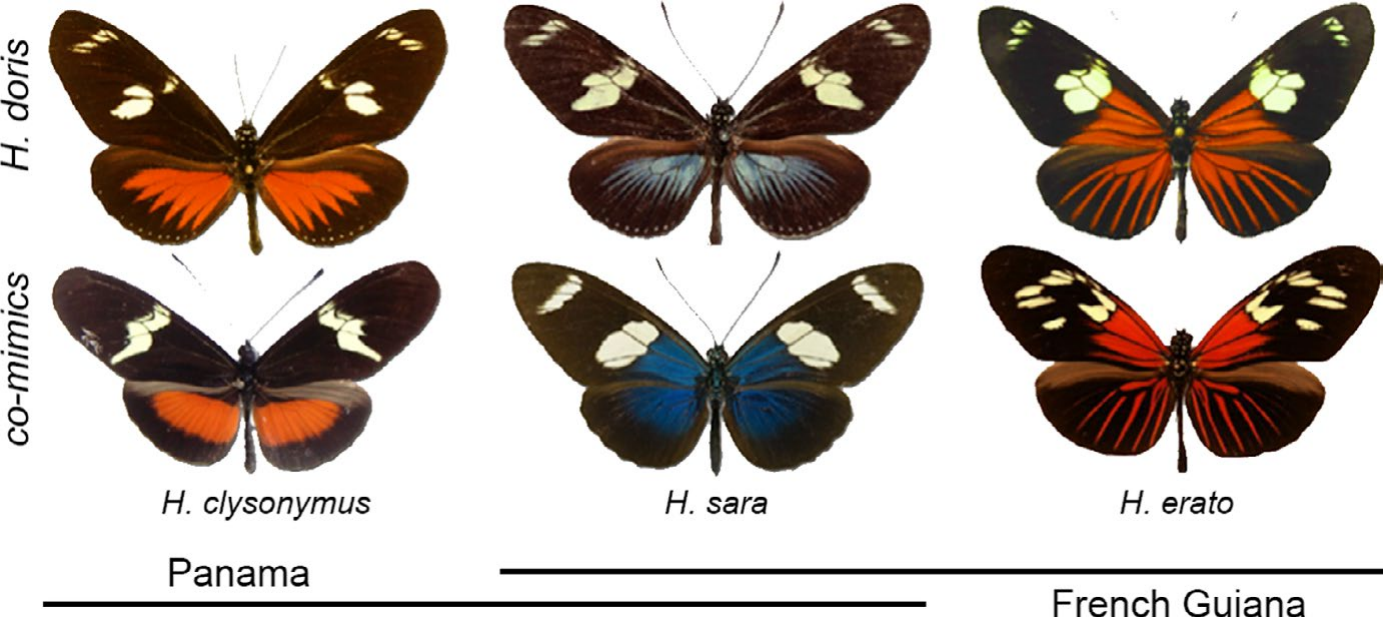
*Heliconius doris* polymorphic forms and co‐mimics. (a) Distribution of blue and divergent red morphs compared to widespread blue morph distribution. (b) *Heliconius doris* morphs (top row) with respective co‐mimics (below)

In this study, with test sites throughout Central and South America, we set out to characterize the ecological pressures that drive polymorphism in aposematic butterflies. The Müllerian mimic *Heliconius doris* is known for being polymorphic across its entire geographic distribution that spreads across most of South and Central America (Constantino et al., 2005; Mallet, 1999), with both red and blue color morphs found throughout its range. While these two morphs are ubiquitous to all *H*. *doris* populations, personal observations point out blue morphs being more abundantly found than red morphs in coastal areas of French Guiana. Additionally, red morphs show a divergence in the red rayed pattern where rays have a broader shape in Central America where red banded co‐mimics are common and thinner rays in South America which perfectly match those of the thin red ray mimicry ring of the amazon basin (see Figure 1). Here, we tested if red and blue morphs of *H*. *doris* reflect predictions of a balanced polymorphism, which we define as a genetic polymorphism that is stable and maintained in a population by natural selection. We, therefore, expected both morphs to experience a similar predation pressure wherever they are both local. We also used the regional color pattern difference in the red *H*. *doris* morphs between South and Central America to assess the ability of pFDS to drive adaptive divergence of a balanced polymorphism at varying geographic scales. Furthermore, we tested if the differences in co‐mimic frequency in French Guiana from rayed phenotypes in the interior to nonrayed in the coast (Blum, 2008), can drive local differences in predation on *H*. *doris* morphs.

Even though Müllerian mimicry theory predicts warning signal monomorphy over time, we have found the selective pressures that allow *H*. *doris* to maintain multiple warning colors across its range. We have further observed how the same selective forces maintaining this polymorphism also act to drive divergence in warning coloration at large geographical scales.

## 2 METHODS AND MATERIALS

### 2.1 Experimental locations

Experiments were conducted at three locations, with two transects at each location. At all sites, *Heliconius* butterflies, particularly the *H*. *doris* co‐mimics *H. erato and H*. *sara*, are some of the most abundant butterflies present (Figure 1). In addition, *H*. *doris* has been observed at each of the three locations. Therefore, the local predators should be well‐trained for avoiding the local morphs. In Panama we conducted the experiments along Pipeline Rd. near Gamboa Panama (9.12542, −79.71459). In Panama, red (broad rays) and blue *H*. *doris* morphs are present, as well as co‐mimics for red and blue morphs. In French Guiana, experiments were conducted in two locations, inland (4.57768, −52.39848) and coastal (4.87316, −52.26627). At the interior French Guiana sites both the red (narrow rays) and blue *H*. *doris* morphs are present, as well as red and blue co‐mimics. At the coastal sites, both the red (narrow rays) and blue *H*. *doris* morphs are present, but only co‐mimics of the blue morph are present. In French Guiana, at around 20 km inland there is a sharp transition in the co‐mimic *H*. *erato* color patterns, with solid black hindwings (nonmimetic to *H*. *doris*) along the coast and red rayed morphs in the interior (mimetic to *H. doris*). (Blum, 2008). Based on personal observations and available collections, *Heliconius* with red ray morphs appear to be largely absent from the coastal areas, therefore, predators in the coastal sites have likely had more training to avoid blue, than red warning color morphs.

### 2.2 Artificial butterfly models

We used artificial butterflies to assay the predation of *H*. *doris* warning colorations in three distinct geographic locations with known divergence in *H*. *doris* wing colorations (Panama versus French Guiana red hindwing pattern). Artificial butterfly experiments in natural populations have proven to be efficient means to record predator attacks for several *Heliconius* species and warning colorations (Arias et al., 2016; Chouteau et al., 2016; Finkbeiner et al., 2018; Seymoure et al., 2018). At each of the three locations in Panama and French Guiana, we used artificial butterflies of three *H*. *doris* warning colorations and the palatable *Pierella hyceta*, which we used as a control following the method in Chouteau et al. (2016). This model allowed us to obtain additional data on the intensity of selection at each locality, however, it also provided a comparative insight on the selection against a palatable phenotype versus an aposematic phenotype.

Standardized photographs of the ventral and dorsal wings of each butterfly were used and printed on two‐sided matte photographic paper. (Epson C135041569 paper and L110 Printer). In order to produce a high volume of standardized models, a silicon mold (Mold Star, Smooth‐on) was fabricated using clay bodies that were shaped to resemble *Heliconius* bodies. The paper wings were inserted into each mold along with a thin 20‐cm metal wire before pipetting a mixture of high melting point wax with a black dye and then left to solidify. The different colors on the printed wings were calibrated in Photoshop (Adobe Inc.) and then contrasted with the colors on actual *H*. *doris* wings by measuring the reflectance spectra of red, black, yellow, and blue using a spectrophotometer (HR2000+ES, Ocean Optics) and a deuterium/halogen light source (DH‐2000; Ocean Optics) connected to a 3.175‐mm diameter sensor (QR600‐7‐UV125BX; Ocean Optics) inserted in a miniature black chamber. Reflectance spectra were taken at 90° for all colors except for the blue structural coloration which was taken at 45° incidence relative to a 99% reflectance standard (300–700 nm; Spectralon) and to a dark current. Spectra were recorded with SpectraSuite 1.0 software (Ocean Optics). Color spectra from real and printed wings were then compared using the method described by Vorobyev and Osorio (1998) in Avicol v.6 software (Gomez, 2006). We contrasted blue, black, red, and yellow, under two main avian vision systems: blue tit (*Parus caeruleus*) for UV vision, with cone proportion and sensitivity as described by Hart et al. (2000), and wedge‐tailed shearwater (*Puffinus pacificus*) as described by Hart (2004) for violet (V) vision. Photoreceptor activity was computed from the Weber fraction (Osorio, 1998), and set to 0.05 for all artificial models. Small gap light conditions, as defined by Endler (1993) from French Guiana were included in all calculations (Thery et al., 2008). Chromatic (Delta S) and achromatic differences (Delta Q) for all colors were found to be under the noticeable threshold for avian vision in UVS and VS (<1.00 Just Noticeable Difference units, as in Llaurens et al. (2017), thereby confirming the accuracy in color of our printed wings to real wings (See Table A1).

Using the attached thin metal wire, models were placed on leaves, trunks, or twigs in visible, well‐lit areas at 10‐m intervals along a 4‐km transect in each site. The placement of each model was carried out so as to mimic the natural perching behavior of *Heliconius* butterflies and provide a visible target for potential avian predators. The distinct model morphs were placed along the transect in a regular order. From 376 to 416 models were placed per site and left for 72 h, after which models were collected. Damage was clearly visible in the malleable wax bodies and paper wings of several models. Damages were catalogued as either (a) “invertebrate attack” when bearing the visible fine marks of arthropod mandibles, often on the wax bodies, (b) “Avian Attack” when bearing the characteristic U or V shape marks on the wax, or (c) “Unknown Predator” when a severe attack was evident, but a specific mark was not found, such as when wings were torn or wax bodies broken in pieces. Models that bore attack marks characteristic of invertebrates were not included in the data analysis (*n* = 97 out of 2271), as there is currently no literature regarding invertebrates carrying the cognitive capacity necessary to make the associations between unpalatability and warning color patterns central to Müllerian mimicry. Furthermore, missing models were also excluded from the analyses as we are unable to determine if they were displaced by falling forest debris, human action, or attacked by natural predators.

### 2.3 Data analysis

Variation in predation rates among the different models in the different regions (consisting of two transects made in the same area), was assessed by a *χ* ^2^ test of independence in R Studio (RStudio Team, 2020). When significant, the Freeman–Tukey deviate (FT) was compared with an alpha, from 0.05 to 0.01, criterion corrected for multiple comparisons using a Bonferroni correction, to identify which model morph was attacked significantly more or less than expected based on the null hypothesis of equal attack probability.

## 3 RESULTS

We placed an average of 392 models per site over 6 sites from a total of 2356 throughout all our field sites of which 2271 were recovered (96.39%) with 158 showing evidence of an attack event (6.96%). In French Guiana, of 1604 model placements throughout 4 field sites, 1524 were recovered (95.01%) and 123 models were attacked (8.07%). In Panama, we placed 752 models throughout 2 field sites of which 747 were recovered (99.34%) and 35 models were attacked (4.69%). Attacks were recorded as damage caused on the wax bodies or paper wings by either avian, unknown, or invertebrate predators.

### 3.1 Balanced polymorphism of aposematic wing colors in *H. doris*

**FIGURE 2:**
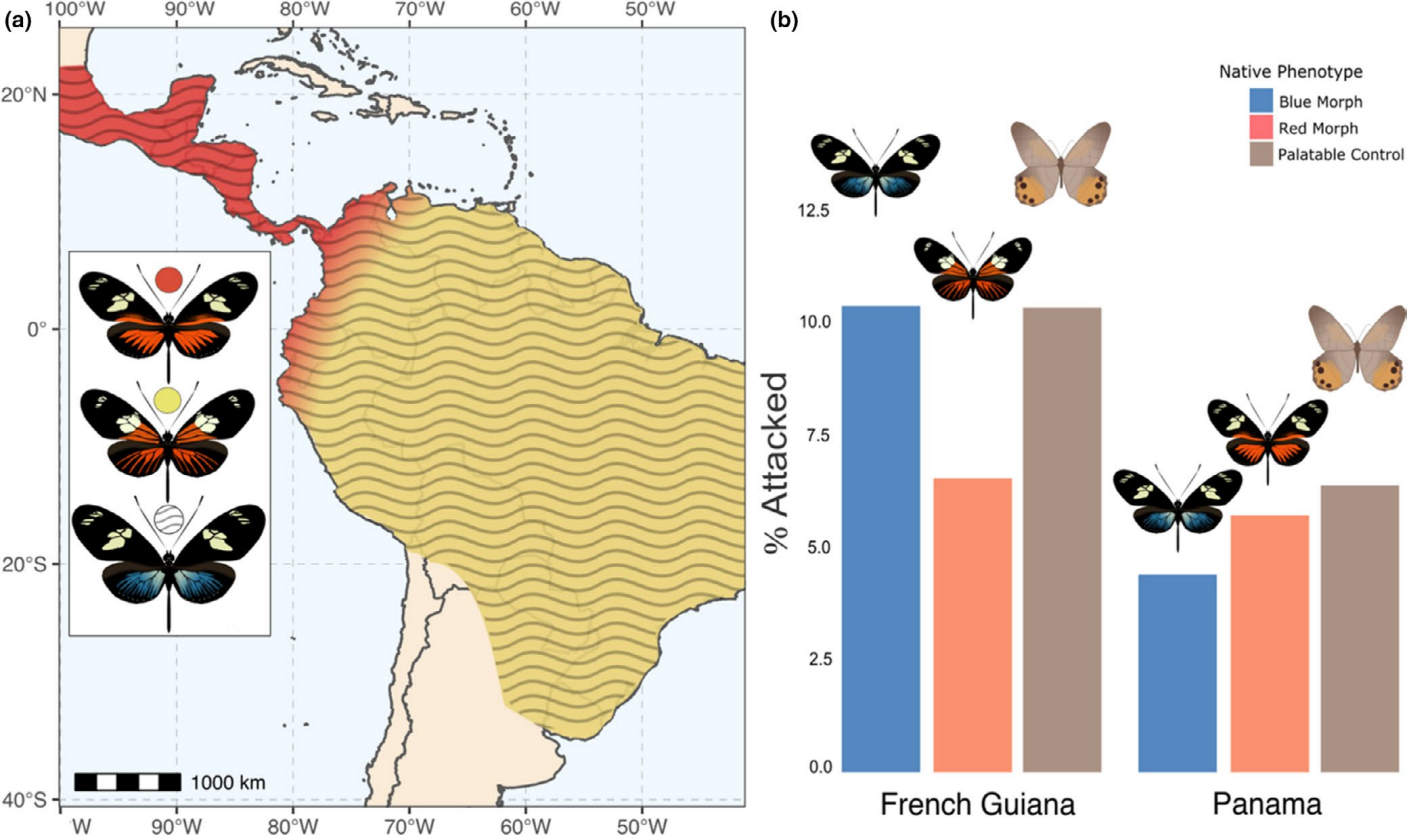
No significant attack differences between *H*. *doris* coexisting morphs. (a) Distribution of *Heliconius doris* morphs. (b) Percentage of attacks on individual models representing local *H*. *doris* morphs in French Guiana and Panama. Statistical analyses used raw attack numbers, percentages shown here for clarity. Bar plots represent two separate FT tests

We tested the prediction that blue and red *H*. *doris* morphs experience similar predation where they are both native. For this we conducted FT tests to determine if there were significant differences in attacks on native blue morphs, native red morphs, and the controls. In French Guiana, using data from all sites, we found no significant differences in attacks (*N* = 1145, *p* > .467). In Panama, we also found no significant differences in attacks between native morphs and controls (*N* = 560, *p* > .306), see Figure 2. This suggests that the blue and red morphs enjoy similar protection from predators wherever they naturally co‐occur in populations. Before pooling data and to confirm that proportions of attacks were consistent between coastal and inland locations in French Guiana, we carried out a *χ* ^2^ test of independence which validated the uniformity of the data (*N* = 1145, *p* > .1).

### 3.2 Regional divergence in balanced polymorphism

Next, we tested for evidence of adaptive divergence of warning coloration among populations for *H*. *doris*. For this, we tested the prediction that divergence in red color morphs between Panama and French Guiana *H*. *doris* populations resulted in greater predation on non‐native red morphs at each locality.

**FIGURE 3:**
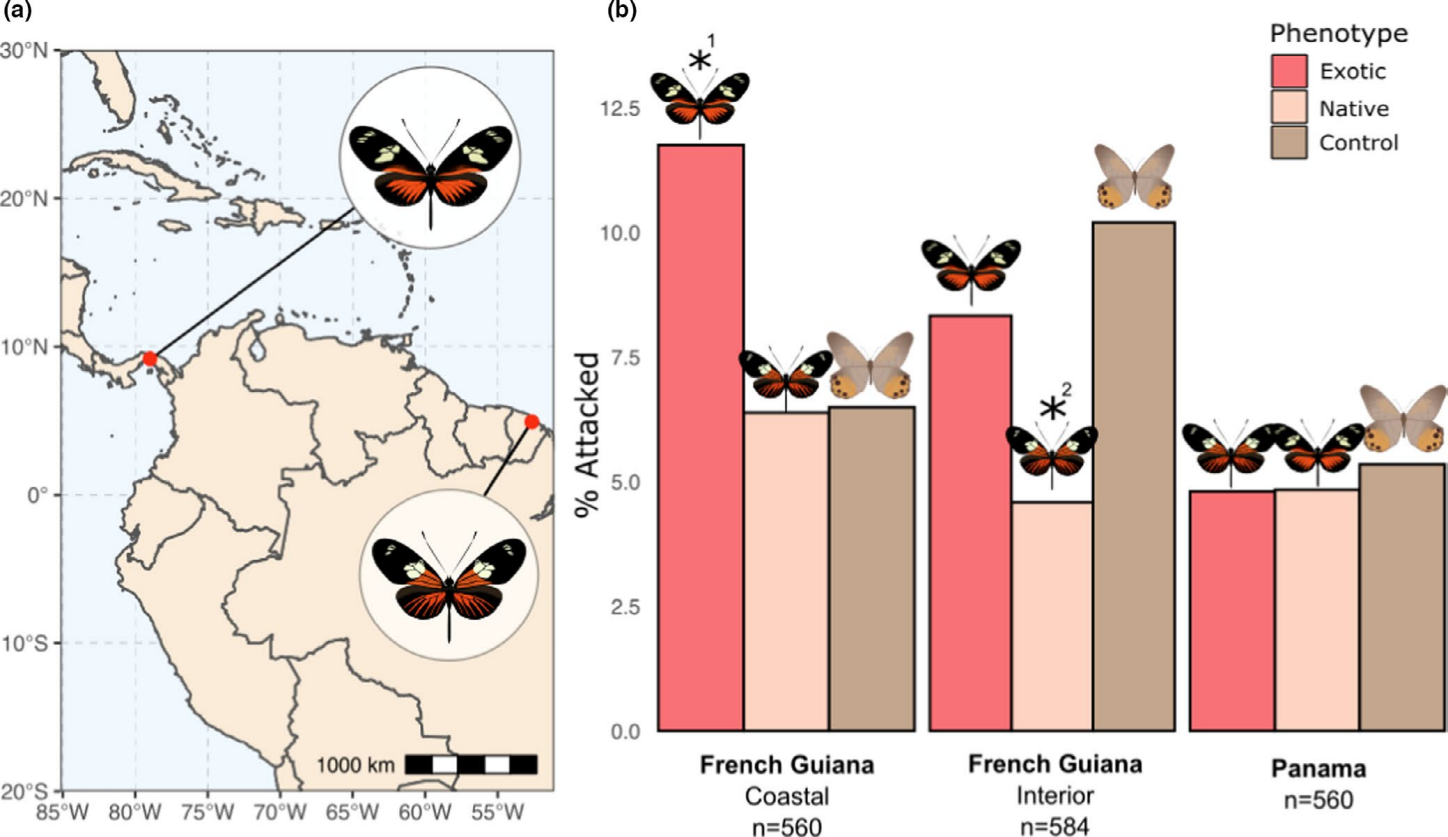
Differences in attacks on native and foreign red morphs. (a) Divergent red morphs of *H*. *doris* at study sites (Panama and French Guiana). (b) Percentage of attacks on native and exotic red phenotypes shows a significantly higher predation on exotic phenotypes in the coastal region of French Guiana and significantly lower predation on native phenotypes in the interior zone, where red co‐mimics are abundant (*^1^ *p* < .0365, *^2^ *p *< .023, Freeman–Tukey [FT] test). Statistical analyses used raw attack numbers, percentages shown here for clarity. Barplots represent two separate FT tests

Of the two red morphs with differing hindwing rays corresponding to Central America or South America, we expected predation rates to show signs of differential avoidance based on frequency of a given signal in each region. Specifically, we predicted that local phenotypes would be significantly avoided relative to the exotic phenotypes. For this test we used FT tests to detect differences in attacks on native, non‐native reds, and controls. In French Guiana, we found significant differences in attacks on red morphs at coastal and inland sites. At coastal sites we found significantly greater attacks on the non‐native red morph than native reds and controls, as would be expected since the local predators would have been naive to this red warning wing pattern (*n* = 560; *p* < .0365; Figure 3). However, at the inland sites the non‐native morphs were not attacked significantly more than the controls. Rather, we found that the native reds were attacked significantly less at inland sites (*n* = 584; *p *< .023; Figure 3). Interestingly, this finding fits the expectations of pFDS, as red co‐mimics are known to be at higher frequencies at inland than in coastal sites in French Guiana. In Panama, we found no evidence of differences in attacks on native reds, non‐native reds, or controls (Table A2).

### 3.3 Co‐mimics drive local variation in pFDS on balanced polymorphism

Lastly, we tested for variation in local selection to explain the geographic differences in the balanced warning color polymorphism in *H*. *doris*. For this, we leveraged differences in the presence of red co‐mimics at coastal versus inland sites in French Guiana. At coastal sites, *H*. *erato* and *H*. *melpomene* morphs are characterized by an all‐black hindwing that lacks red rays. However, at inland sites, *H*. *erato* and *H*. *melpomene* morphs have red rays that are strikingly similar to *H*. *doris* red rays. At coastal and inland sites, *H*. *sara*, which is a co‐mimic of the blue warning color, is found at high frequencies. Similar to the analyses above, we used FT tests to determine if attacks were different on red morphs, blue morphs, or controls, in either coastal or inland sites. We predict that pFDS would result in red morphs being attacked more at coastal sites that lack the red co‐mimics.

We found no evidence of red morphs being attacked significantly more than blue morphs or the controls at the coastal sites (*n* = 557; *p* > 1.0). However, at the inland sites we found that the red morphs were attached significantly less than blue morphs and controls (*n* = 588; *p* < .027). These results suggest that the presence of co‐mimics confers greater protection for red morphs, however, a lack of co‐mimics does not appear to result in greater predation pressures for the red morphs.

## 4 DISCUSSION

### 4.1 Striking similarity of predation pressures across *Heliconius* species and populations

Our results of predator attacks on *Heliconius* models correspond to the attack patterns observed in mark–release–recapture experiments, where naïve predators significantly attack novel, exotic patterns relative to native, common warning patterns (Langham, 2004; Mallet & Barton, 1989). As may be expected, attacks of live prey showed much greater differences for exotic and native morphs (36–37% vs. 0%, respectively), than we observed with artificial models (5–11.5% vs. 4–6.5%, respectively; see Figure 3). This difference between model and live prey experiments likely results from the inability of models to replicate physical and behavioral cues recognized by potential predators (i.e., crawling, wing flapping, flight). Although the attack numbers on models likely do not reflect realized predation rates of live butterflies, they do provide reliable means to compare relative predation pressures due to differences in visual cues.

Importantly, there is a remarkable similarity in attacks of *Heliconius* models in studies that span a variety of species and geographic locations over the past decade (Arias et al., 2016; Chouteau et al., 2016; Finkbeiner et al., 2012, 2014, 2018; Merrill et al., 2012; Seymoure et al., 2018). Attack percentages in these studies range from 4% to 15%, which overlaps our observed attack percentages that ranged from 4% to 12% on *H*. *doris* morphs. This consistency in attacks of models may reflect the similarity in avian predation pressures among *Heliconius* species and populations. The rufous‐tailed jacamar (*Galbula ruficauda*) has been reported as a common predator of *Heliconius* in western South America (Mallet & Barton, 1989) and Central America (Dell’Aglio et al., 2016; Langham, 2004). In line with these reports, we observed a rufous tailed jacamar successfully attack a *Heliconius* (likely *Heliconius sara*) at one of our inland experimental sites in French Guiana. Collectively, this supports that jacamars may be a common predator driving similar attack rates in the various *Heliconius* model experiments. Therefore, we can make direct comparisons among these studies and general inferences about the relative effectiveness of specific aposematic color patterns and corresponding selective pressures.

For studies measuring the effectiveness of FDS, abundancy data of the organisms being investigated can be useful for better understanding training of local predator populations. In organisms such as *Heliconius* butterflies, such abundance data can be quite difficult to collect and interpret. For example, species such as *H*. *erato* and *H*. *sara* tend to be quite abundant and broadly dispersed across their ranges, while species such as *H*. *doris* tend to be very localized and their local densities can vary dramatically between generations. This is likely due to life‐history differences among the co‐mimics, with *H*. *doris* being a species where multiple females lay eggs in aggregate and gregarious larval feeding that tends to fully consume local host plants, causing the next generation to disperse to find new suitable host plants. Existing collection records lack the locality and color pattern details to inform us of *H*. *doris* local abundances. The difficulties in acquiring such abundance data highlight the usefulness of predation experiments such as that presented here in assessing FDS pressures.

### 4.2 The paradox of polymorphic mimicry in *H. doris*

The strong selective forces that drive Müllerian mimicry are predicted to result in monomorphism among mimicking species, yet as in *H*. *doris*, there are many examples of polymorphic mimicry in nature. Our study sheds some light on how this paradox may be achieved. Our results suggest that pFDS can vary at regional scales, and is constrained to knowledgeable predator communities which are savvy to the aposematic forms found only in their local ecosystem (Chouteau et al., 2016; Langham, 2004). For example, over the relatively short distance of ~30 km, we found significant differences in the attacks on native red morphs of *H*. *doris*, with significantly less attacks occurring at the sites where other red co‐mimics are present. This suggests the predator community knowledge was quite distinct at the different sites and corresponds to reports of jacamars having rather narrow home ranges (Chai, 1986). However, this begs the question of “how do the red *H*. *doris* persist in areas lacking red co‐mimics?,” as we would expect the lack of co‐mimics to result in higher predation and eventual removal of the red morph from the population.

A possible explanation lies in the dispersal behavior from nearby populations where red co‐mimics are present and the red *H*. *doris* morphs have greater protection. Other *Heliconius* species such as *H*. *erato* and *H*. *melpomene* have an estimated dispersal range of only ~2.5–5 km (Mallet, 1986a; Mallet et al., 1990), as a result of their “trap‐line” behaviors as adults (Young & Montgomery, 2020). However, it has been suggested that *Heliconius doris* may disperse much larger distances immediately post pupal eclosion, which could reduce chances of sib‐competition and sib‐matings (Mallet, 1999). *Heliconius doris* females are known to gather in groups and lay eggs on single plants, often even the same leaf, which we observed firsthand in French Guiana. This results in a mass of gregarious larvae that will often fully consume all leaves and tendrils on the *Passiflora* host. After consumption, an individual host plant can require several years to reach a size sufficient to host another population of *H*. *doris* eggs. It would then likely benefit newly eclosed females to disperse larger distances than other *Heliconius* species that tend to oviposit much fewer eggs in close proximity. Therefore, it is possible that group egg laying, and relatively greater dispersal in *H*. *doris* could drive a “mismatch” of warning colors in the distribution of *Heliconius* co‐mimetic species, as seen in French Guiana. This dispersal‐based hypothesis would result in sink populations for *H*. *doris* morphs, where the red co‐mimics are lacking, that are continuously replenished from source populations where red morphs have greater protection. It is difficult to understand how this could be an evolutionarily stable strategy, and dispersal data for *H*. *doris* are lacking to support such a source‐sink model for the presence of red *H*. *doris* morphs where the co‐mimics are absent.

Another important aspect that could explain the distribution of red morphs and polymorphic mimicry in *H*. *doris* is the genetic basis for the color variation. In *H*. *numata*, polymorphic color patterns result from allelic changes at a single locus, *P* (Joron et al., 2006). More specifically, the different color patterns result from varying combinations of chromosomal inversions across the *P* locus (Joron et al., 2011). The color pattern variation is maintained in local populations through disassortative mating (Chouteau et al., 2017; Maisonneuve et al., 2021), a form of negative frequency‐dependent selection where rare morphs are preferred mates resulting in offspring of variable colorations. Since the color pattern differences are controlled by a single locus, and the different alleles cannot recombine due to the inverted orientations (Jay et al., 2021), disassortative mating will keep producing color pattern variation in perpetuity. In *H*. *numata*, each of the different color patterns also corresponds to local co‐mimics, and different morphs appear to share similar predation pressures (Chouteau et al., 2016). We propose that a similar system may have evolved in *H*. *doris*, with nonrecombining alleles at a single locus controlling color pattern variation coupled with disassortative mating as such a system would result in distinct red and blue morphs in each generation across the *H*. *doris* range. The lack of intermediate phenotypes encountered among *H*. *doris*, lends support to such a genetic architecture for this polymorphism as if it were controlled by multiple unlinked genes, we would expect to find mismatched recombinants and nondistinct morphs (Jamie & Meier, 2020), which are extremely rare and slight in *H*. *doris*.

Alternatively, it is also possible that polymorphism in *H*. *doris* may be maintained without the need of chromosomal inversions or a supergene type architecture, just as it is maintained in *Heliconius cydno* through positive FDS (Davey et al., 2017; Kapan, 2001). This can especially be so in a butterfly such as *H*. *doris* that can lay many eggs in a single plant and whose gregarious larvae can result in a large single brood where the frequency of a rare, exotic phenotype can be high enough to train local predators. In this case, the high frequency of individuals with an exotic pattern would influence the local mimetic signal, and polymorphism could be maintained. Currently, there are no data for the inheritance of color patterns or mate preference in *H*. *doris*, which would be vital for determining how polymorphic mimicry is maintained in the species.

### 4.3 Positive FDS as an agent of convergence and divergence

**FIGURE 4:**
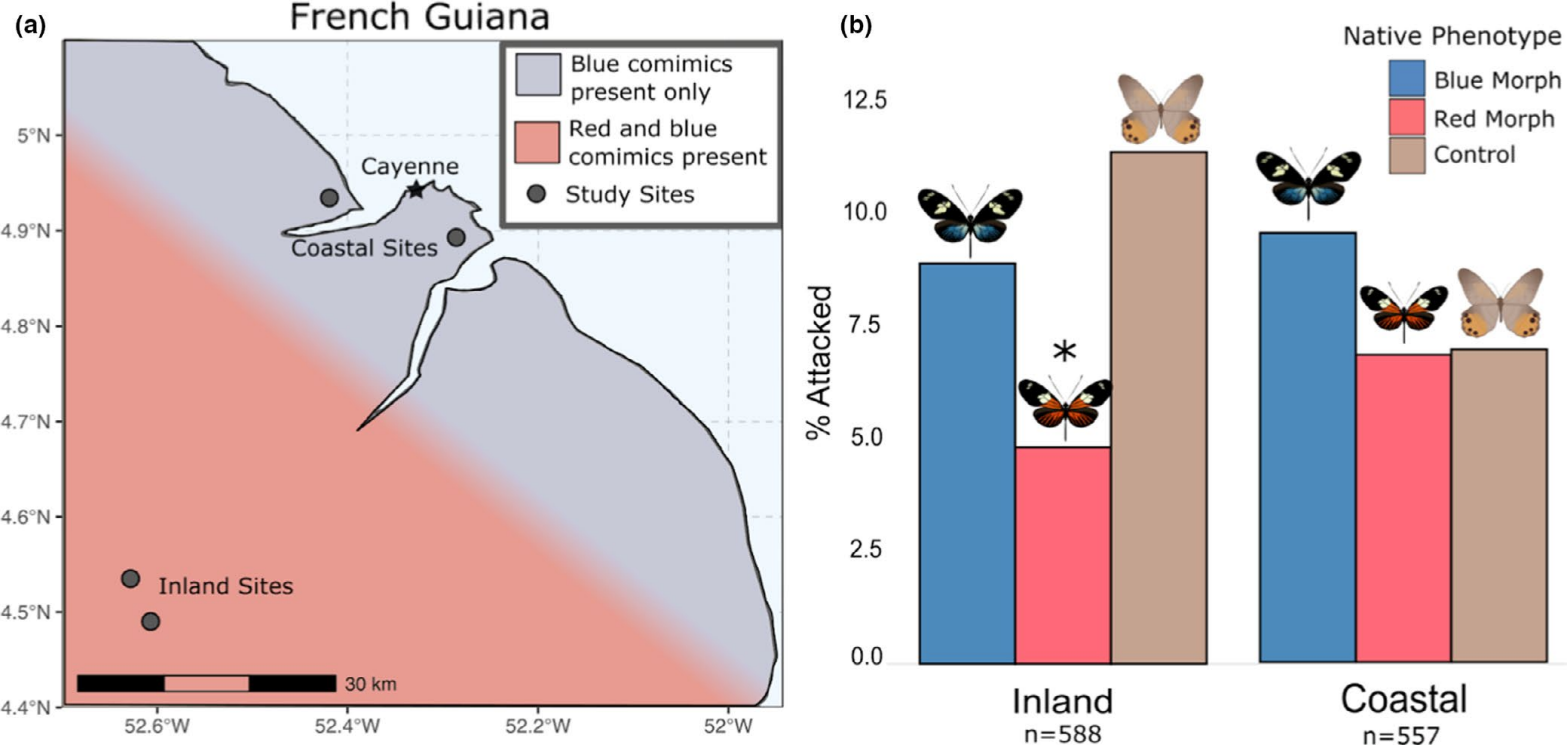
Red morphs attacked less at sites with red mimicry ring present. (a) Locations of study sites and co‐mimic distributions in French Guiana. (b) Attacks of different *H*. *doris* morphs and controls at inland and coastal sites that differ in local mimicry rings show significant protection of red morphs at inland sites where red co‐mimics are present (*p < .027, Freeman–Tukey test). Statistical analyses used raw attack numbers, percentages shown here for clarity. Barplots represent two separate FT tests

Positive FDS is the evolutionary force that drives mimicry in *Heliconius* butterflies (Chouteau et al., 2016). It is the result of local predators learning through experience to avoid the aposematic signals of the most common unpalatable prey. Müllerian mimicry posits that unpalatable prey will benefit by sharing similar aposematic signals thereby allowing them to share the cost of training the local prey population. As we saw in our study, local pFDS can be a strong evolutionary force that can vary over relatively short distances. Within *Heliconius* populations, pFDS will drive mimics to a local optima color pattern that often varies little within or between species. In our study, this is clearly seen in the French Guiana red morphs whose hindwing rays are near perfect copies of the hindwing rays of *H*. *erato*, the most common *Heliconius* in French Guiana with a red rayed color pattern. In contrast, the blue *H*. *doris* are co‐mimics of *H*. *sara*, which do not have blue *rays*, but rather a blue iridescence that extends broadly from the proximal region of the forewings (Figure 1). Correspondingly, the shape of the blue rays of *H*. *doris* are starkly different from the shape of the red rays. Therefore, not only has pFDS driven a difference in hindwing color, but also the shape of the color pattern. This is further seen in Panama, where again the red *H*. *doris* morphs are shaped differently than those in French Guiana, where the red ray shape is a near perfect to red co‐mimics in Panama (Figure 1). This variation in color pattern shape exemplifies the power of pFDS to drive convergence (or advergence) within local populations (Figure 4).

In contrast to our results indicating local advergence, the difference in color pattern shape between regions demonstrates the ability of pFDS to drive divergence within species. Across its range, *H*. *doris* presently exhibits divergent red color pattern morphs, that in French Guiana were distinguishable by the local predator community.

In Panama, local predators attacked the models much less and did not show evidence that they distinguished between the native and exotic morphs. We suggest this may be a result of a more generalized avoidance in Panama, which would result in less attacks on all morphs, as we saw. A similar difference in prey discrimination between South and Central America has also been noted by Finkbeiner et al. (2018). Additionally, our Panama transect has been the location of many *Heliconius* studies (live and model based) in recent years (Dell’Aglio et al., 2016; Finkbeiner et al., 2014; Merrill et al., 2012; Seymoure et al., 2018), this could also explain predator avoidance of artificial models if these had an earlier exposure to artificial butterfly models. Panama is also a known *Heliconius* hybrid zone (Mallet, 1986b). Therefore, local predators often encounter intermediate phenotypes which makes precise pattern recognition a nonviable strategy for avoiding distasteful prey. Thus, it may benefit potential predators to adopt general avoidance of aposematic wing colors (red, black, yellow, blue) and flight behaviors common of unpalatable prey.

Alternative explanations for the regional differences in predation pressures involve variation in local prey composition, mimicry rings and chemical defenses. Regional differences in prey composition and abundance have been previously shown to impact predation. For example, predators can show higher rates of avoidance of both models and imperfect mimics when alternative prey is abundant (Kokko et al., 2003; Lindström et al., 2004). Differences in mimicry ring compositions and densities between South American and Central America could certainly also contribute to the regional differences we observed in selection pressures. This could be exacerbated by regional differences in differences in toxicity, which is known for several *Heliconius* species (de Castro et al., 2021; Mattila et al., 2021; Sculfort et al., 2020). Current data show little variation in toxicity of *H*. *doris* from Panama to Peru (Sculfort et al., 2020). However, *H*. *doris* shows higher toxicity than its co‐mimics *H*. *sara* and *H*. *erato* in Panama, relative to South America. This suggests *H*. *doris* predation in Panama may be lower due to the higher unpalatability of co‐mimics but a more in‐depth study on predation which included co‐mimic toxicity data would be needed.

We found that even at small regional scales, selection on mimetic warning patterns differs depending on local predator communities. Although Müllerian mimicry theory predicts mimicking species to achieve monomorphism in color patterns over time, we demonstrate that *H*. *doris* maintains a balance of multiple warning colors across its range. Furthermore, we find that the same selective forces acting to maintain the balanced polymorphism, also drive divergence in warning coloration across its range. These results highlight the complex nature of pFDS and the impacts it has on interspecific variation of mimetic warning colorations. Collectively, our study as well as other model studies, such as those of color polymorphisms in *H*. *numata* (Chouteau et al., 2016) and Peruvian dart frogs (Chouteau & Angers, 2011), have demonstrated that pFDS can simultaneously be an agent that both limits and facilitates diversification of mimetic traits.

## CONFLICT OF INTEREST

The authors declare no competing interests.

## AUTHOR CONTRIBUTIONS

**James G. Ogilvie:** Conceptualization (equal); data curation (lead); formal analysis (lead); investigation (lead); methodology (equal); project administration (equal); writing – original draft (lead); writing – review & editing (equal). **Steven Van Belleghem:** Data curation (equal); formal analysis (equal); writing – original draft (equal); writing – review & editing (equal). **Mathieu Chouteau:** Conceptualization (equal); data curation (equal); formal analysis (equal); funding acquisition (lead); investigation (equal); methodology (lead); resources (equal); software (lead); writing – original draft (supporting); writing – review & editing (equal). **Ryan Range:** Data curation (equal); funding acquisition (equal); supervision (equal); writing – review & editing (equal). **Riccardo Papa:** Conceptualization (equal); funding acquisition (equal); writing – review & editing (equal). **Owen W. McMillan:** Investigation (equal); resources (equal); supervision (equal); writing – review & editing (equal). **Brian A. Counterman:** Conceptualization (equal); data curation (equal); funding acquisition (lead); investigation (equal); methodology (equal); project administration (equal); resources (lead); supervision (lead); writing – original draft (supporting); writing – review & editing (equal).

### OPEN RESEARCH BADGES

This article has been awarded Open Materials, Open Data Badges. All materials and data are publicly accessible via the Open Science Framework at https://datadryad.org/stash/share/DUZo3NWNy9XuslH7sGEvhrvy2zrjx5qBPgvJkCco60U.

## Data Availability

Data generated from this study and R‐scripts utilized can be accessed at the Dryad Digital Repository https://doi.org/10.5061/dryad.h9w0vt4j5.

## APPENDIX A

### Attacked models

(a) Model displaying characteristic invertebrate minute jaw marks distributed throughout the body (common on models placed on cecropia trees which are often inhabited by colonies of *Azteca* ants). (b) Unknown vertebrate predator attack (or mixed predators). (c) Avian predator attack (damage usually concentrated on anterior or posterior extremities of the main body). (d) Avian attack showing peck marks.

**TABLE A1:** Chromatic (Delta S) and achromatic differences (Delta Q) for all colors were under the noticeable threshold for avian vision in UVS and VS (<1.00) Just Noticeable Difference units, confirming the accuracy in color of our printed wings to real wings

Black	VS		UVS	
	DeltaS	DeltaQ	DeltaS	DeltaQ
	0.779014	0.665228	0.904758	0.679434
Yellow	VS		UVS	
	DeltaS	DeltaQ	DeltaS	DeltaQ
	0.864394	0.30895	0.754856	0.619833
Red	VS		UVS	
	DeltaS	DeltaQ	DeltaS	DeltaQ
	0.834949	0.291156	0.541547	0.27452
Blue	VS		UVS	
	DeltaS	DeltaQ	DeltaS	DeltaQ
	0.845082	0.587016	0.754856	0.619833

**TABLE A2:** Total attacks by avian and unknown predators (invertebrate attacks excluded), in all study sites in French Guiana and Panama

French Guiana	Panama Red Morph	Blue Morph	French Guiana Red Morph	Control
Coastal sites (red co‐mimics absent)				
Site 1	12	12	9	8
Site 3	10	4	3	4
Interior sites (red co‐mimics present)				
Site 2	8	13	7	15
Site 4	8	3	2	5
Panama				
Site 5	5	3	4	6
Site 6	4	4	5	4
